# Multivalent and synergistic chitosan oligosaccharide-Ag nanocomposites for therapy of bacterial infection

**DOI:** 10.1038/s41598-020-67139-7

**Published:** 2020-06-19

**Authors:** Lin Mei, Zhenlong Xu, Yanmei Shi, Chunlei Lin, Shuyan Jiao, Lijun Zhang, Pengxu Li

**Affiliations:** 1 School of Materials and Chemical Engineering, Zhongyuan University of Technology, Zhengzhou, 450007, P.R China; 2 Scientific Research Center, Henan University of Chinese Medicine, Zhengzhou, 450046, P.R China; 3 College of International Education, Zhongyuan University of Technology, Zhengzhou, 450007, P.R China

**Keywords:** Biomedical materials, Biomedical materials

## Abstract

Chitosan oligosaccharide functionalized silver nanoparticles with synergistic bacterial activity were constructed as a multivalent inhibitor of bacteria. Placing the chitosan oligosaccharide on silver nanoparticles can dramatically enhance the adsorption to the bacterial membrane via multivalent binding. The multicomponent nanostructures can cooperate synergistically against gram-positive and gram-negative bacteria. The antibacterial activity was increased via orthogonal array design to optimize the synthesis condition. The synergistic bacterial activity was confirmed by fractional inhibitory concentration and zone of inhibition test. Through studies of antimicrobial action mechanism, it was found that the nanocomposites interacted with the bacteria by binding to Mg^2+^ ions of the bacterial surface. Then, the nanocomposites disrupted bacterial membrane by increasing the permeability of the outer membrane, resulting in leakage of cytoplasm. This strategy of chitosan oligosaccharide modification can increase the antibacterial activity of silver nanoparticles and accelerate wound healing at the same time. The nanomaterial without cytotoxicity has promising applications in bacteria-infected wound healing therapy.

## Introduction

Human health has been threatened with abuse of antibiotics and the appearance of multiple drug-resistant bacteria^1,2^. In the European Union, the number of deaths attributable to antibiotic-resistant bacteria is about 25,000 each year^3^. Therefore, it is crucial to design and develop new antimicrobial materials with high antibacterial activity and low resistance in the field of biomedicine.

Silver nanoparticles (AgNPs) with small size and large specific surface area have a stronger antibacterial effect against different bacteria, virus, and fungi^4–6^. AgNPs can contact with bacterial cell membranes, penetrate into the cytoplasm, and then inactivate essential respiratory enzymes and proteins, leading to bacterial death^7,8^. However, these antibacterial action of AgNPs is often dependent on high concentration since their physical collision with bacterial surface is random^9^. For enhancing interaction of AgNPs and the bacteria, cationic polymers-stabilized AgNPs were designed to bind to the negatively charged cell surfaces of the bacteria by electrostatic interaction^10,11^. However, the toxicity of cationic polymers obstructed their biomedicine application. Then, to improve their biocompatibility, collagen-stabilized AgNPs were prepared by a photochemical method^12^. However, it was found that this AgNPs showed a weaker antibacterial property, comparing with small molecules-stabilized AgNPs (eg. lysine and citrate).

Chitosan oligosaccharide (COS), which is a cationic, non-immunogenic, biocompatible and an FDA-recognized mucoadhesive polymer^13^, with low molecular and good water-solubility is the positively charged alkaline-amino-oligosaccharide. Several biological activities of COS, such as hemostasis, antibacterial activity and anti-inflammatory effects, have been extensively studied in biomaterials field^14^. Conjugation with COS to the surface of AgNPs can increase their surface charge and enhance the adsorption to the negatively charged bacterial cytoplasmic membrane through electrostatic interactions. Moreover, COS as antimicrobial agent in multivalent binding can mediate the penetration of AgNPs into the bacteria^15,16^. To the best of our knowledge, COS-functionalized AgNPs (AgNPs-COS) as multivalent inhibitors of the bacteria has not been reported.

In this study, a green and simple method has been developed for the preparation of AgNPs-COS as a novel antimicrobial nanomaterial. AgNPs-COS can be achieved via surface-modification *in situ* by reduction of AgNO_3_ in the presence of COS. To achieve highly efficient antimicrobial ability, the synthesis conditions of AgNPs-COS are optimized by orthogonal array design (OAD). The fractional inhibitory concentration (FIC) and zone of inhibition test are used to characterize synergistic antimicrobial activity. Furthermore, effect of metal ions, the outer membrane permeabilization assay and scanning electron microscopy (SEM) is performed to explore antibacterial action mechanism. Finally, we further explore the biocompatibility toward RAW 264.7 cells and the treatment of bacteria-induced wound infection.

## Materials and methods

### Materials

COS was purchased from TCI Chemical (Tokyo, Japan). Silver nitrate (AgNO_3_, 99.995%, metals basis, Ag 63% min), citric acid (CA) and 1-N-phenylnaphthylamine (NPN) was obtained from Aladdin Chemical Reagent (Shanghai, China). *E. coli* ATCC 25922 and *S. aureus* ATCC 25923 strains were provided by College of Life Science of Zhengzhou University (Zhengzhou, China). All solutions were prepared by the ultrapure water and stored in the refrigerator at 4 °C.

### Synthesis and characterization of AgNPs-COS

Briefly, under vigorous stirring, 500 µL of 1.0% (*w/w*) AgNO_3_ and 500 µL of 1.0% (*w/w*) COS were added into 48 mL ultrapure water and then heated to 80 °C. 900 µL of 1.0% (*w/w*) CA solution was rapidly added and kept stirring for 30 min. The obtained solution (dark yellow) was dialyzed (8–14 kDa M.W. cutoff) by the ultrapure water for 12 h and stored at 4 °C for use.

The ultraviolet-visible (UV-vis) absorption spectra of the samples were recorded on a UV-vis spectrophotometer (Shimadzu, UV-2700) using quartz cell. The morphology of obtained nanoparticle images was observed by a transmission electron microscope (TEM, FEI TECNAI G2 F20) operating at 200 kV.

### Minimal inhibitory concentrations (MIC)

The bacteria were grown overnight in Luria-Bertani (LB) broth at 37 °C and then diluted with LB broth to approximately 2.0 × 10^6^ CFU/mL. The bacterial suspension was mixed in equal volume of two-fold diluted COS, AgNPs or AgNPs-COS solution and incubated for 8 h at 37 °C. The bacterial suspensions used for MIC determination with the antibacterial agent (COS, AgNPs or AgNPs-COS solution) were mixed during incubation. The inhibited visible growth of bacterial cells was assessed by determining an optical density at 600 nm (OD_600_) measured by UV-vis spectrophotometer. Each assay was carried out in triplicate.

### Zone of inhibition test

After overnight incubation at 37 °C, the bacterial suspensions were diluted with LB broth and grew to approximate 5.0 × 10^6^ CFU/mL. 50 μL of the bacterial suspensions were swabbed onto the surface of LB agar plates. Then the circular blotting paper (diameter 1.0 cm) with 100 μL COS, AgNPs or AgNPs-COS solution (at the same concentration) was gently placed in the middle of the LB agar plates, and incubated for 6 h at 37 °C. The antibacterial activity was measured by evaluating the zone diameter around the disk.

### Effect of Mg^2+^ ions on the antibacterial activity

The bacterial suspension was centrifuged at 6000 rpm for 5 min and washed with phosphate buffer solution (PBS, 0.01 mol/L, pH 7.4) three times. The supernatant was removed and the remaining bacteria were resuspended in PBS. 20 µL of MgCl_2_ solution was added to 1.5 mL of bacterial suspensions (approximate 1.0 × 10^7^ CFU/mL) to a final concentration of 0.1 mmol/L. Then, 100 µL of AgNPs-COS solution (the final concentration of 8.0, 4.0, 2.0, and 1.0 μg/mL) was added to the suspensions and incubated at 37 °C for 30 min. 1.6 mL of LB broth was added to the mixtures and incubated at 37 °C for another 8 h. The OD_600_ was measured using UV-vis spectroscopy.

### Outer membrane permeabilization assay

*E. coli* cultures were harvested by centrifugation at 6000 rpm for 5 min, and rinsed with PBS three times. The final bacteria were resuspended in 1.5 mL PBS (approximate 1.0 × 10^7^ CFU/mL). Then 50 µL of 1.0 mg/mL NPN and AgNPs-COS solution with different final concentrations were added into the above bacterial suspension. The fluorescence intensity was determined by fluorescence spectrometer (Hitachi, F-7100). An excitation wavelength was 350 nm and an emission wavelength was 370–550 nm. The control assay was performed by adding PBS instead of AgNPs-COS.

### Preparation of bacterial sample for SEM

The bacterial suspension (approximate 4.0 × 10^7^ CFU/mL) was washed with PBS and resuspended in 1.5 mL PBS. Then, 100 µL of 64 μg/mL AgNPs-COS was added for 10 min. Free AgNPs-COS was removed by centrifuging at 6000 rpm for 5 min, washing by PBS three times and resuspending in 0.5 mL PBS. The treated bacteria were placed on the poly-ɛ-lysine coated glass slide and fixed with 2.5% glutaraldehyde overnight. After removing the excess glutaraldehyde solution and washing with water for three times, the remaining bacterial was dehydrated with a series of ethanol aqueous solutions (30, 50, 70, 90, 95, 100% v/v) for 10 min successively and dried in a freeze dryer. The sample was glued to the aluminum stud with double-sided adhesive conductive tape. After treated with platinum spraying, the bacterial morphology was observed using a field emission SEM (FEI Quanta 250 FEG).

### Cytotoxicity test

The RAW 264.7 cells were obtained from Henan University of Chinese Medicine (Zhengzhou, China). The cells were cultured in DMEM medium (Gibco) supplemented with 10% fetal bovine serum in 5% CO_2_/95% air at 37 °C. The cells were seeded into 96-well plates at a density of 10^4^ cells per well and further incubated for 24 h. Then the medium in the wells was replaced with the different concentrations of AgNPs-COS solution. After incubation for 24 h, 10 µL of 5% MTT solution was added into each well and further incubated for 4 h. After removing the medium containing MTT, 100 µL of DMSO was added into each well to dissolve the formazan crystals with low-speed shaking for 15 min. The OD_570_ value was measured using a microplate reader. The untreated cells were used as positive control.

### *In vivo* tests

Male Wistar rats (weighing 190–210 g) were offered from Henan University of Chinese Medicine (Zhengzhou, China). Animal welfare and experimental procedures were strictly performed in accordance with the Guidelines for Animal Experimentation of Henan University of Chinese Medicine (Approval Number: DWLL2018030038) and the protocol was approved by the Animal Ethics Committee of Henan University of Chinese Medicine.

The rats were anaesthetized with intraperitoneal injection of chloral hydrate and then shaved the hair on the backs. A full thickness cutaneous wound within a defined 1.0 cm i.d. circular area was made and then injected with 50 µL bacterial suspension (*S. aureus*, approximate 2.0 × 10^7^ CFU/mL) by a pipette tip. The bacterial suspension was evenly applied to the wound surface to ensure that the bacteria was only spread on the wound. After modeling success, there were the secretions of yellowish pus from infected tissue. The rats were divided into four groups and each group had six rats. Daily therapy was administered 50 µL 8.0 µg/mL AgNPs-COS, 75% ethanol, and 0.9% NaCl solution to the wounds of three groups once a day, respectively. The wounds of the fourth group were untreated as the blank control. After 12 days, the skin tissues in contact with the samples were collected for hematoxylin and eosin (H&E) staining for histopathology evaluation and Collagen I immunohistochemical analysis by a Rat Collagen I Immunohistochemistry Kit.

The quantitative real-time polymerase chain reaction (qRT-PCR) was used to analyze the relative expression levels of the various genes. The reaction was performed by a Fluorescent Quantitative PCR Detector (7000 Fast, ThermoFisher) and SYBR Green I qRT-PCR Master Mix (MCE) was used. For the specificity of the amplification in the reactions, the melting curve and gel electrophoresis were used in assessment of qRT-PCR products. The condition of qRT-PCR cycling was 95 °C for 2 min, 95 °C for 10 s, 60 °C for 30 s, 40 cycles. The relative expression level of each gene was calculated by the 2^−ΔΔCq^ method. Primers used for the qRT-PCR analysis of the expression levels of Collagen I and other genes were listed in Supplementary Table S1 online.

## Results and discussion

### Orthogonal array design for optimizing the synthesis conditions of AgNPs-COS

AgNPs-COS were synthesized via reduction of AgNO_3_ by CA at 80 °C in the presence of COS in water. The amine groups of COS can complex with silver cations and then conjugated on the growing AgNPs surface following reduction process. The antibacterial activity of AgNPs-COS was related to the amount of AgNO_3_, COS, and CA in synthesis process. The amount of AgNO_3_ and CA was found to be related to the size of AgNPs-COS and then can affect the antibacterial activity of AgNPs-COS. COS as capping groups to the AgNPs surface can stabilize AgNPs and then affect the surface plasmon resonance absorbance of AgNPs. OAD can quickly generate useful information on key variable by arranging different factors within a single experiment. The results of OAD can then be analyzed by analysis of variance. Optimization of synthesis condition via an OAD would reduce the amount of experiments and costs^17,18^. Therefore, OAD was used to optimize experimental conditions of AgNPs-COS. In this study, the synthesis conditions include three factors: the amount of AgNO_3_, COS, and CA. Therefore, an OAD L_9_ (3^4^) was used to evaluate effects of these factors. Each factor was evaluated in three levels. Experiments were carried out with 1.0% (*w/w*) AgNO_3_ solution at 300, 500, or 700 µL, 1.0% (*w/w*) COS solution at 200, 500, or 800 µL, and 1.0% (*w/w*) CA solution at 300, 600, or 900 µL, respectively. The OAD experiments were performed according to Table 1. Subsequently, the MIC values against *S. aureus* and *E. coli* were determined to evaluate the antibacterial activity of the nine synthesized AgNPs-COS and the results were shown in Tab. 1. Clearly, AgNPs-COS synthesized by the optimum synthesis conditions (No.5 in Table 1) can obtain the lowest MIC value and act synergistic antibacterial functions against gram-positive (*S. aureas*) and gram-negative bacteria (*E. coli*). Therefore, the optimum conditions were 500 µL of 1.0% (*w/w*) AgNO_3_ solution, 500 µL of 1.0% (*w/w*) COS solution, and 900 µL of 1.0% (*w/w*) CA solution, respectively. Under this condition, synthesized AgNPs-COS achieved the desired antimicrobial activities toward both gram-positive and gram-negative bacteria.

**Table 1:** Factors and levels for L_9_ (3^4^) OAD experiments and MIC for AgNPs-COS against *S. aureas* and *E. coli*.

No.	Amount of	Amount of	Amount of	MIC against	MIC against
	AgNO_3_ (μL)	COS (μL)	CA (μL)	*S aureas* (μg/mL)	*E coli* (μg/mL)
1	300	200	300	2.56 ± 0.49	1.83 ± 0.65
2	300	500	600	1.98 ± 0.12	1.64 ± 0.35
3	300	800	900	1.42 ± 0.22	0.96 ± 0.24
4	500	200	600	1.56 ± 0.55	1.11 ± 0.40
5	500	500	900	0.78 ± 0.26	0.51 ± 0.18
6	500	800	300	1.21 ± 0.43	0.82 ± 0.26
7	700	200	900	1.78 ± 0.47	1.35 ± 0.27
8	700	500	300	1.82 ± 0.53	1.37 ± 0.45
9	700	800	600	1.19 ± 0.30	0.97 ± 0.33

### Characterization of AgNPs-COS

**Figure 1:**
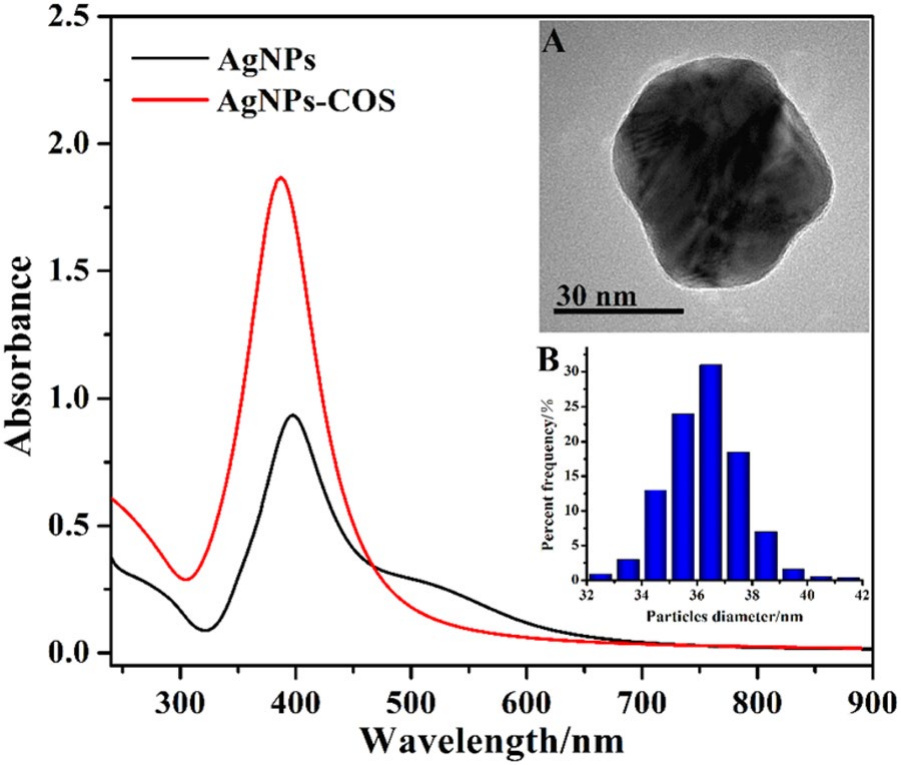
UV-vis spectra of AgNPs and AgNPs-COS. Inset: TEM image (**A**) and size distribution (**B**) of AgNPs-COS.

UV-vis absorption spectrum was used to confirm the formation of AgNPs-COS. The spectra of AgNPs and AgNPs-COS were shown in Fig. 1. Compared with the absorption spectrum of AgNPs, the maximum absorption wavelength of AgNPs-COS showed a blue shift from 400 to 391 nm. Moreover, the peak intensity of AgNPs-COS was higher than that of AgNPs. COS as stabilizers was beneficial to the nucleation and growth of nanoparticles and avoided the formation of these large nanoparticles. In addition, the surface charge analysis was performed. The zeta potential of AgNPs was −20.3 mV. Although AgNPs with a large number of negative charges can maintain stability in aqueous solution, the interaction between AgNPs and the bacteria is impeded by electrostatic repulsion. However, the zeta potential of AgNPs-COS was 11.3 mV, which could enhance their adsorption to negatively charged bacterial membranes by electrostatic interaction. This result indicated that COS molecules successfully binding to the AgNPs surface.

The morphology of AgNPs-COS was observed by TEM (Fig. 1, inset A). It can be found that the obtained nanoparticles were spherical with good dispersibility and COS coated on AgNPs surface in the core-shell module. The average diameter of AgNPs-COS was 36.7 nm (Fig. 1, inset B). Because of conjugation of COS onto AgNPs surface, the edge of AgNPs-COS emerged light corona (Fig. 1, inset A) compared with AgNPs (see Supplementary Fig. S1 online).

### Study of synergistic antimicrobial activity

The FIC index was employed to detect any synergistic antimicrobial effect between AgNPs and COS by a two-dimensional microdilution assay. The FIC was calculated as follows:

$${\rm{FIC}}=\frac{{\rm{MIC}}\,{\rm{of}}\,{\rm{compound}}\,{\rm{A}}\,{\rm{in}}\,{\rm{combination}}}{{\rm{MIC}}\,{\rm{of}}\,{\rm{compound}}\,{\rm{A}}\,{\rm{alone}}}+\frac{{\rm{MIC}}\,{\rm{of}}\,{\rm{compound}}\,{\rm{B}}\,{\rm{in}}\,{\rm{combination}}}{{\rm{MIC}}\,{\rm{of}}\,{\rm{compound}}\,{\rm{B}}\,{\rm{alone}}}$$

If the FIC index was less than 0.5, the interaction was defined as synergistic effect^19^. The results showed that the FIC indices were 0.34 and 0.29 against *S. aureus* and *E. coli*, respectively, indicating that COS binding to the AgNPs can obtain synergistic antimicrobial effect.

**Figure 2:**
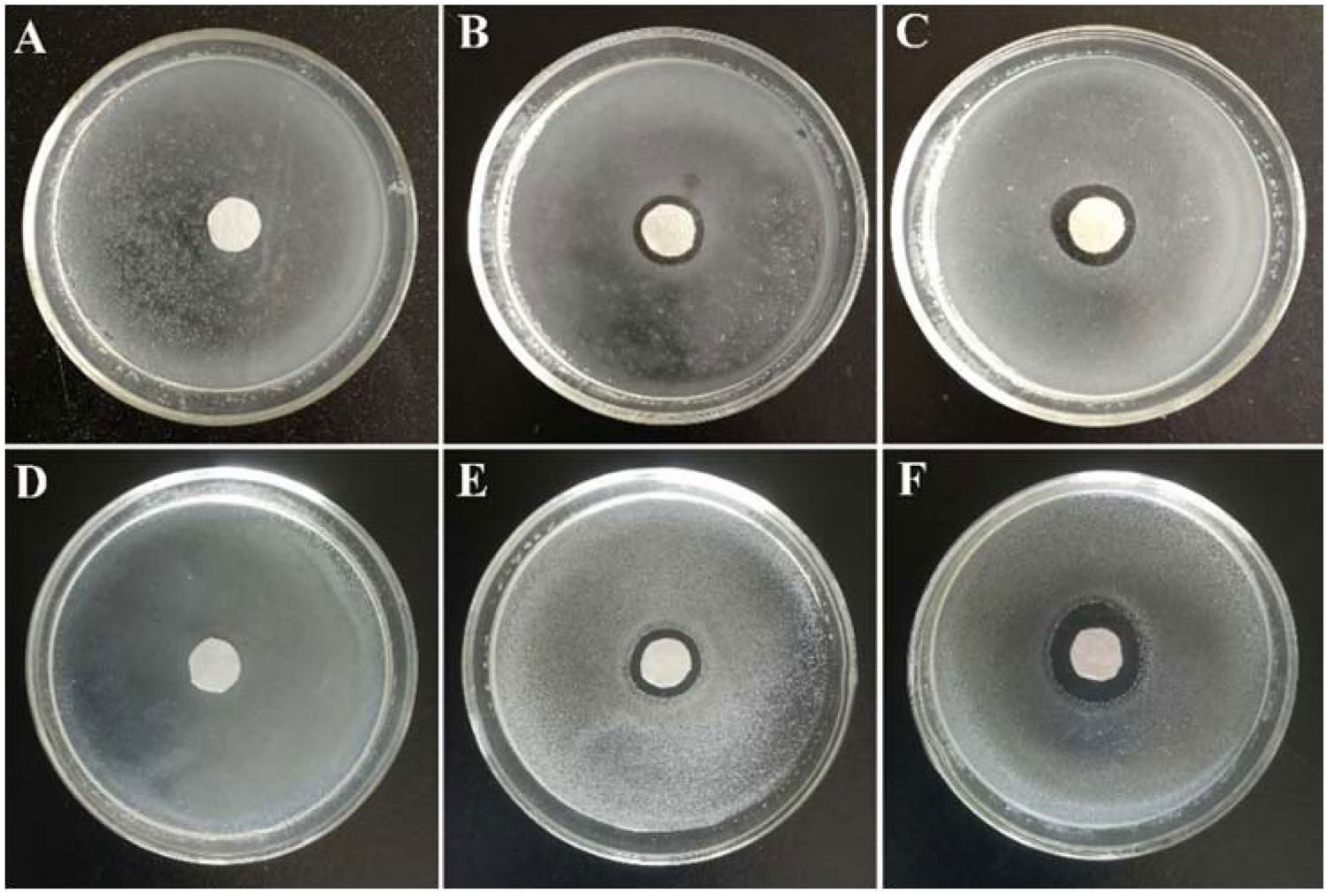
Inhibition zones of COS (**A**,**D**), AgNPs (**B**,**E**) and AgNPs-COS (**C**,**F**) against *S. aureus* (**A**–**C**) and *E. coli* (**D**–**F**).

The synergistic effects between AgNPs and COS was also verified by zone of the inhibition test. For this purpose, COS, AgNPs, and AgNPs-COS at a final concentration of 100, 64, and 64 μg/mL, respectively, was prepared. From Fig. 2, the diameters of zone of inhibition of AgNPs-COS were significantly larger than those of COS and AgNPs, indicating that AgNPs-COS had better antimicrobial performance compared with COS and AgNPs. The outstanding synergistic activity of COS and AgNPs was also confirmed against both gram-negative and gram-positive bacteria. Moreover, COS was used as stabilizers to protect AgNPs against agglomeration for retaining their diffusivity and enhancing antimicrobial property.

### Effect of Mg^2+^ ions on the antibacterial activity of AgNPs-COS

**Figure 3:**
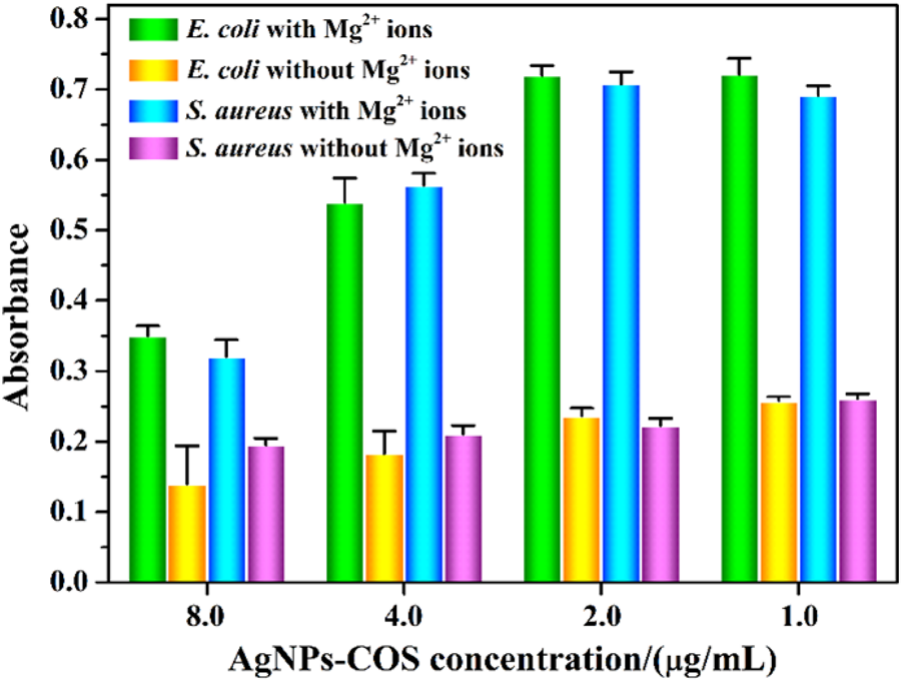
Effect of Mg^2+^ ions (the final concentration of 0.1 mmol/L) on the antibacterial activities against *E. coli* and *S. aureus* with different concentration.

To explore the binding sites of AgNPs-COS on the bacterial surface, the effects of Mg^2+^ ion on the bacterial growth inhibition were examined. From Fig. 3, OD_600_ value of *S. aureus* and *E. coli* were obviously increased when Mg^2+^ ions were added to the bacterial suspensions in the presence of AgNPs-COS with different concentration. It was indicated that the antimicrobial activity of the nanoparticles decreased. It has been reported that the lipopolysaccharide (on the surface of the gram-negative bacteria, e.g. *E. coli*) linked with Mg^2+^ ions via electrostatic interaction to form a stable structure on the surface of the bacterial membrane^20,21^. However, amino group of COS can chelate Mg^2+^ ions by metal-to-ligand π-bonding. Then the lipopolysaccharide was isolated and dispersed to the medium, result in the damage of the outer membrane of the bacteria. When Mg^2+^ ions were added to the bacterial suspension, the COS of the nanoparticle surface would chelate Mg^2+^ ions in the medium, thereby avoiding replacement of these ions from their binding sites in lipopolysaccharide. Similarly, in gram-positive bacteria (e.g. *S. aureus*), the teichoic acids of the bacterial cell wall can attract Mg^2+^ ions to provide rigidity to the cell wall^22^. When COS of the nanoparticle surface chelated Mg^2+^ ions from these original sites on the bacterial surface, teichoic acid would be dispersed to the medium and the bacterial cell wall would be damaged. Therefore, AgNPs-COS can interact with the bacteria by binding to Mg^2+^ of the bacterial surface.

### The permeability of outer membrane

**Figure 4:**
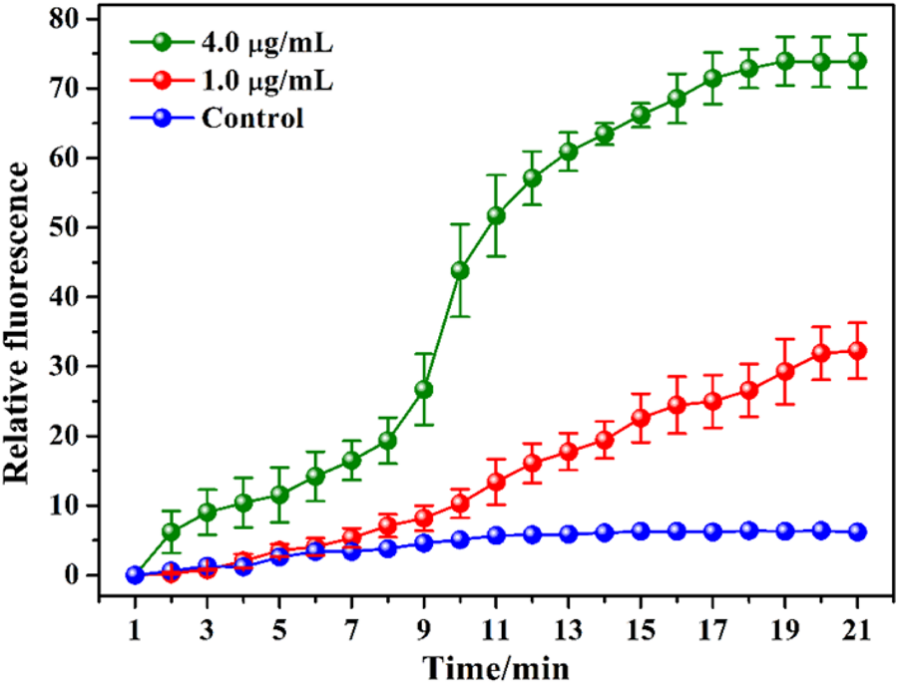
Change of NPN fluorescence intensity with different times and AgNPs-COS concentrations.

The interaction of AgNPs-COS with outer membrane of *E. coli* cells was studied using the hydrophobic fluorescent probe NPN, which has strong fluorescence in hydrophobic environments and weak fluorescence in aqueous environments^23,24^. When AgNPs-COS disorganized outer membrane of bacterial cell, NPN could partition into the phopholipid layer of the outer membrane, which can increase NPN fluorescence intensity. As shown in Fig. 4, NPN fluorescent intensity in *E. coli* suspensions was increased with the increase in the nanoparticles concentration and interaction time. The fluorescent intensity reached a plateau after approximately 20 min, which indicated that AgNPs-COS can permeabilizate the cell membrane and destroy the integrality of bacterial cell.

### Morphological change of the bacteria by SEM observation

**Figure 5:**
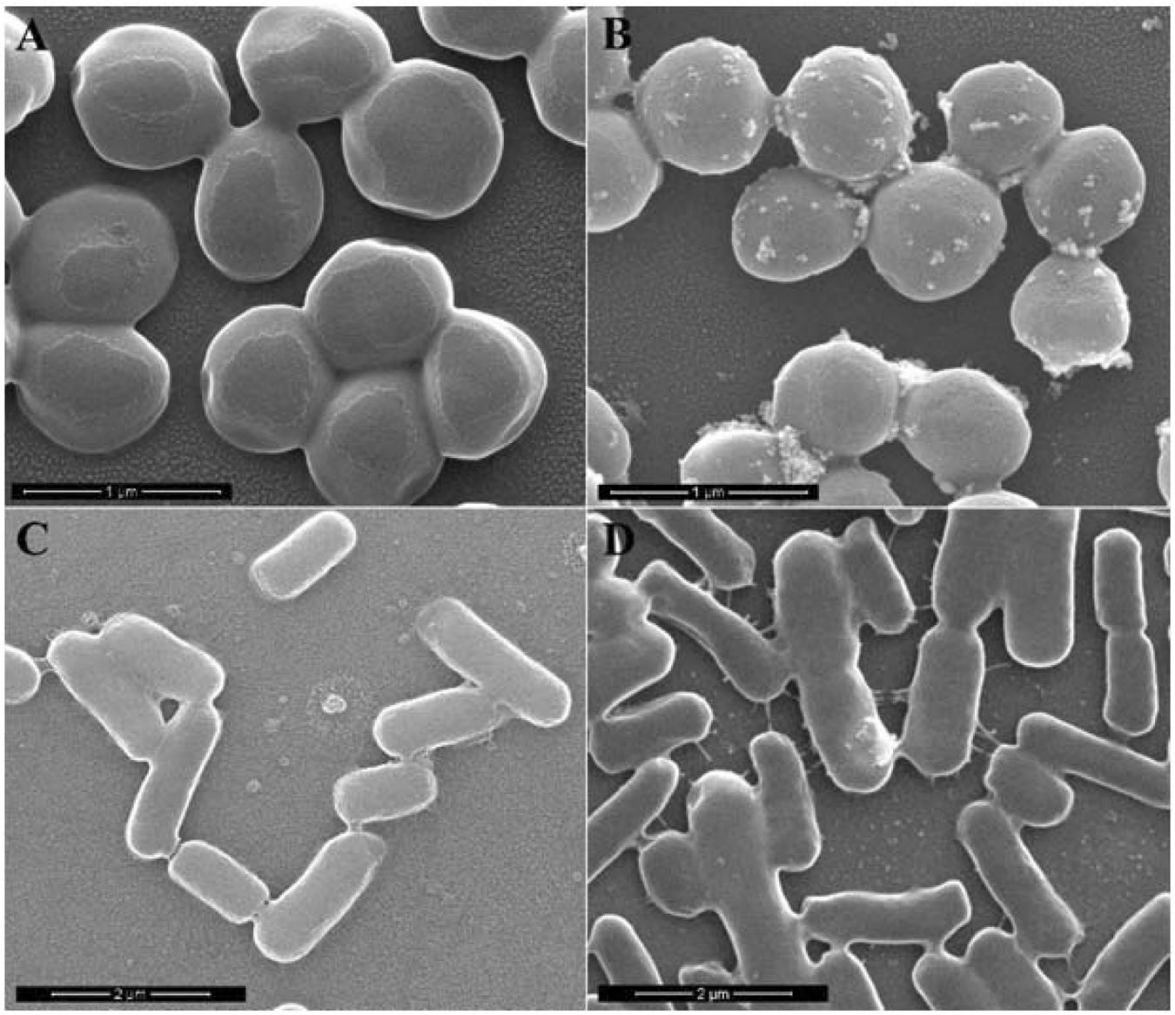
SEM images of *S. aureus* (**A,B**) and *E. coli* (**C,D**) before (**A,C**) and after (**B,D**) being treated with AgNPs-COS for 10 min.

The morphological change of *S. aureus* and *E. coli* before and after incubation with AgNPs-COS were investigated via SEM observations. Untreated *S. aureus* and *E. coli* had a smooth surface with the integrity of membrane structure (Fig. 5A,C). In contrast, the morphology of the treated bacteria is altered significantly after incubation with 4.0 µg/mL AgNPs-COS for 10 min (Fig. 5B,D). The cell walls of *S. aureus* had formed a large number of vesicles (Fig. 5B). Furthermore, the leakage of large cytoplasmic components can be observed on the cytomembrane of *E. coli* (Fig. 5D). Similar SEM images of various bacteria had been also observed in that of the treated bacteria^25–27^. The thickness of the peptidoglycan layer of gram-positive and gram-negative bacteria was difference. When the bacteria interacted with AgNPs-COS for 10 min, *S. aureus* with the thick peptidoglycan layer formed the vesicles (small leakage of cytoplasmic components) and *E. coli* with the thin peptidoglycan layer represented large leakage of cytoplasmic components. It indicates that AgNPs-COS can disrupt the bacterial membrane, leading to leakage of cytoplasm. Then the damaged membrane can destroy the structural integrity and the membrane ability, resulting in bacterial death.

The antimicrobial mechanisms of AgNPs-COS are possibly as follows. AgNPs-COS interact with the bacteria by binding to Mg^2+^ ions of the bacterial surface. Then, the nanoparticles disrupt bacterial membrane via increasing the permeability of the outer membrane, resulting in leakage of cytoplasm. These are possibly reasons causing bacterial cell death.

### Cytotoxicity assay

**Figure 6:**
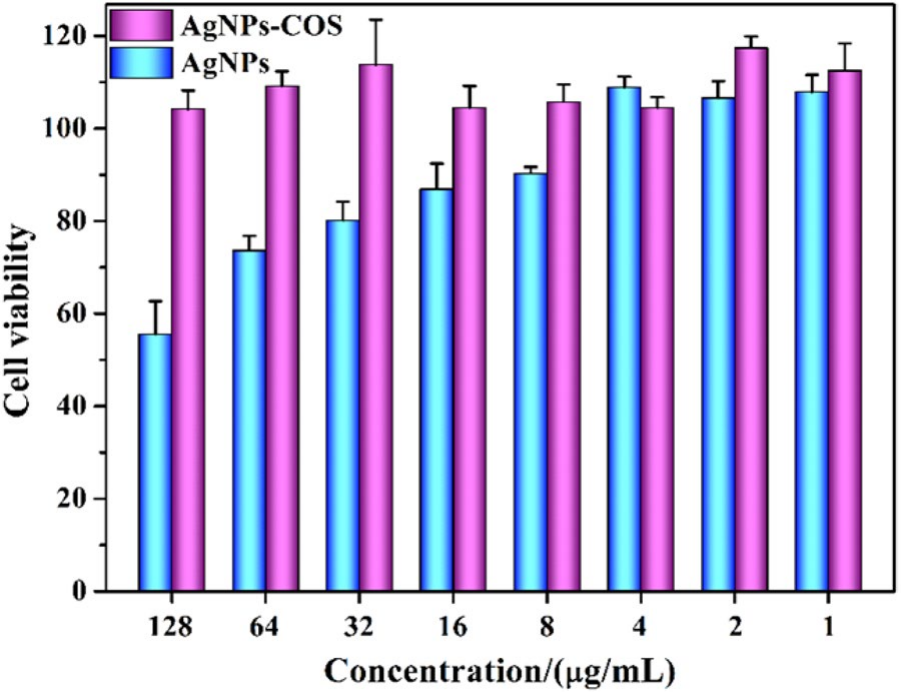
Cell viability after incubation as a function of AgNPs and AgNPs-COS concentrations for 24 h determined by MTT assay.

The cytotoxicity of AgNPs-COS was investigated in Raw246.7 cells by MTT assay. As shown in Fig. 6, no obvious cytotoxicity was observed at a concentration up to 128 μg/mL, which had exceeded 160-fold and 250-fold MIC against *S. aureus* and *E. coli*, respectively (Table 1, No.5). Moreover, the toxicity of AgNPs can be improved by binding to the COS with good biocompatibility. Therefore, the nanoparticles had the potential for *in vivo* use.

### *In vivo* study

**Figure 7:**
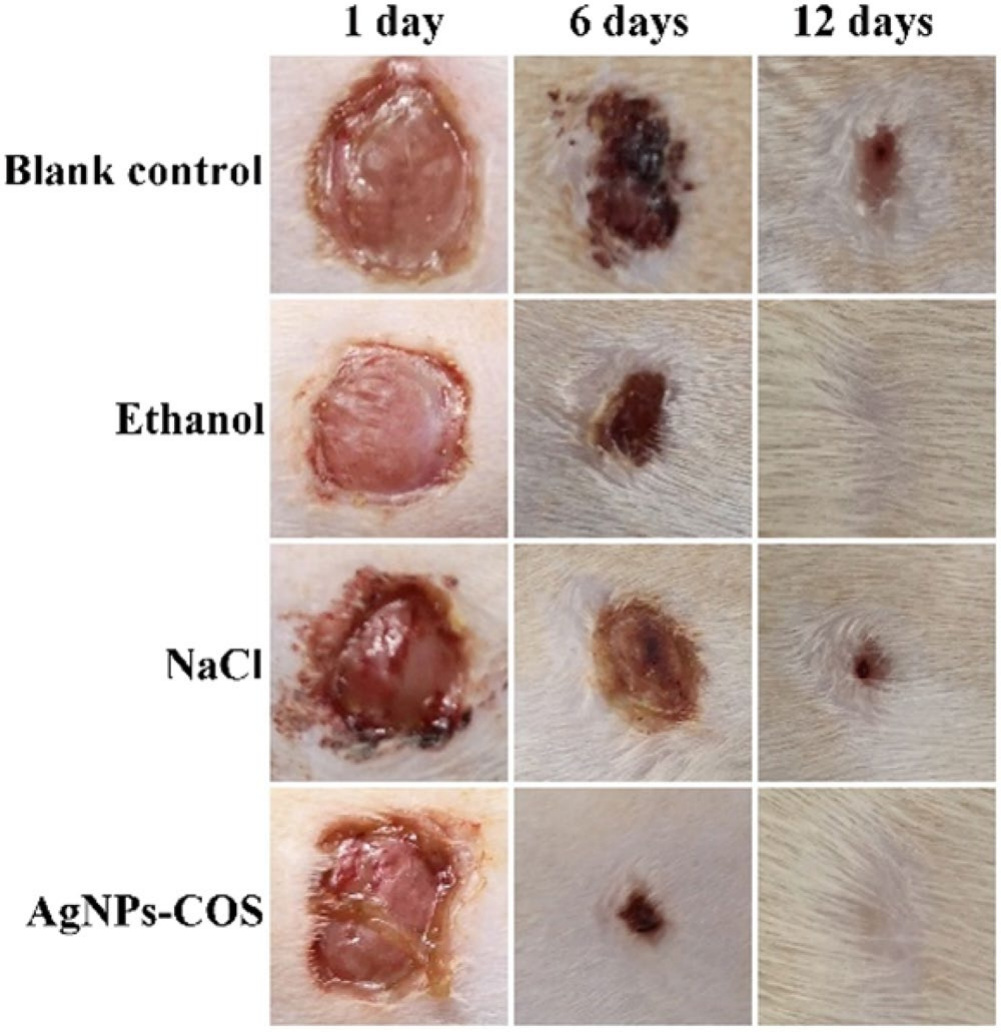
Wound photographs of the rats with the different treatment for 1 day, 6 days and 12 days, respectively.

**Figure 8:**
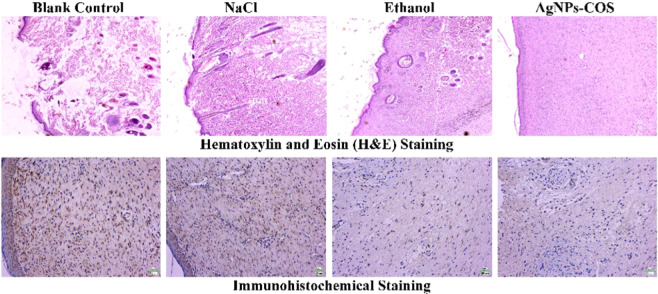
Representative optical micrographs (20×) by H&E staining and immunohistochemical staining of rat skin with the different treatment.

**Figure 9:**
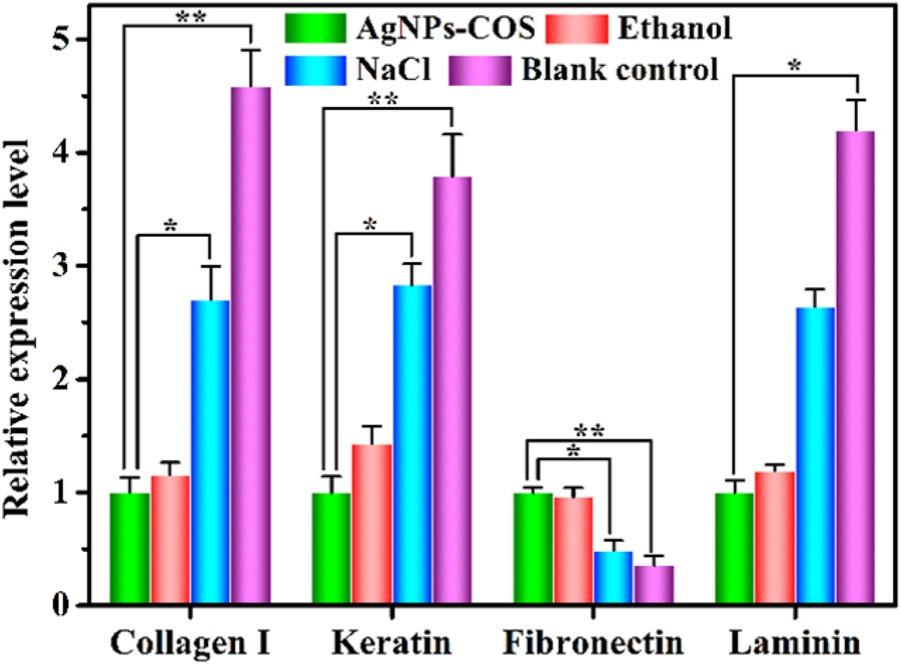
Relative expression levels of collagen I, keratin, fibronectin and laminin in the rat skin tissues. (The error bars indicate means ± SD (n = 3); ^*^P < 0.05, ^**^P < 0.01).

To explore the healing status of the wound infection with *S. aureus*, 8.0 µg/mL AgNPs-COS, 75% ethanol or 0.9% NaCl solution were administered to the wounds of every group for daily therapy, respectively. The physical measurement of wound area before the treatment and during the treatment were evaluated by wound photographs. From Fig. 7, the wounds of AgNPs-COS group and ethanol group were healed after 12 days. However, the wounds treated with NaCl and without treatment were not healed. Histological evaluation of rat dermal wound was performed at the 12th days after treatment and representative optical micrographs by H&E staining were showed in Fig. 8. The epithelialization underlying wound connective tissues were observed by the therapy of AgNPs-COS or ethanol. Meanwhile, a few inflammatory cells emerged from the 75% ethanol-treated wounds. However, massive inflammatory cells appear from the 0.9% NaCl solution-treated and untreated wounds. From immunohistochemical staining of rat epidermal tissues, NaCl group and blank control group had more collagen I (brown parts or dots) than ethanol group and AgNPs-COS group. Moreover, as shown in Fig. 9, relative expression levels of collagen I were significantly increased in the rat skin tissues of NaCl group and blank control group, indicated inflammation still exists. Meanwhile, the results of relative expression levels of keratin, fibronectin and laminin illustrated that AgNPs-COS can promote wound healing of bacterial infection without inflammation^28–31^.

## Conclusions

AgNPs-COS was synthesized as a new antimicrobial material via surface-modification *in situ*. These antibacterial activity could be improved via OAD optimization of synthesis condition. This nanoparticles with synergistic antimicrobial activities could efficiently inhibit the growth of gram-positive and gram-negative bacteria. The studies of antimicrobial mechanism indicated that AgNPs-COS could combine with Mg^2+^ ions of the bacterial surface, interact with the bacterial membrane and increase the permeability of the outer membrane. These interactions will lead to the damage of bacterial membrane and the death of the bacteria. This antimicrobial material has a great potential for the wound healing of damaged skin tissues accompanied with bacterial infection, and will be widely applicable for combating multiple bacteria-induced infectious diseases in medical field.
